# Both the Survival Scenario and the Death Scenario Improve Memory Recall Regardless of the Processing/Priming Paradigm

**DOI:** 10.3389/fpsyg.2018.00793

**Published:** 2018-05-28

**Authors:** Xiaolin Zhao, Hao Li, Xinxin Zhang, Juan Yang

**Affiliations:** ^1^Faculty of Psychology, Southwest University, Chongqing, China; ^2^Key Laboratory of Cognition and Personality, Ministry of Education, Southwest University, Chongqing, China

**Keywords:** adaptive memory, survival scenario, death scenario, processing paradigm, priming paradigm

## Abstract

Memory researchers have suggested human's memory system can help us remember adaptive information conducive to survival and avoiding death. However, in previous studies, the “survival-” orienting task and the “death-” orienting task were adopted in different paradigms. Specifically, the survival-related task was adopted in a processing paradigm, in which participants were instructed to process words in terms of its relevance of survival value, while the death-related task was adopted in a priming paradigm, in which participants were first placed in a death-salient state, and then rated the pleasantness of each word without encoding its death value. The current study aimed to explore whether death scenarios improve recall as much as survival scenarios regardless of the processing/priming paradigm. In Experiment 1, we compared a survival scenario, a death scenario and a control scenario in both processing and priming paradigms. Our results showed that: (a) both survival-related thoughts and death-related thoughts could improve memory recall, both in processing and in priming paradigms; and (b) participants' proportion of correct recall did not show difference between the survival and the death conditions. In Experiment 2, we used a more detailed control scenario and showed that both the death scenario and the survival scenario yielded higher recall than the control scenario in the priming paradigm. Together, our results suggest that both survival and death scenarios have a similar effect on memory recall regardless of the processing/priming paradigms.

## Introduction

Over the past century, evolutionary psychologists have addressed such questions as why our memory systems evolve and if functional properties from our ancestral past still play a role (Bruce, 1985; Sherry and Schacter, 1987; Nairne et al., 2007, 2008; Burns et al., 2011). Indeed, there is now substantial empirical evidence showing that memory is enhanced when people process information in terms of its fitness value, e.g., helping us to find nutrition, protecting us from predators, mating with somebody, living longer, and increasing the opportunity to transmit our genes to the next generation (Klein et al., 2002). For example, Nairne et al. (2007) were motivated by this a priori prediction, based on an evolutionary analysis that memory retention favors the adaptive content of information. In their experiments, participants undertook a processing task in which they were asked to make judgments about the relevance of words to a specific scenario; participants consistently showed the best memory when words were rated for survival, compared with a variety of control tasks (e.g., moving or pleasantness judgment). Their results suggested that our memory systems might have evolved to favor survival-relevant information.

Given that any organism's chances of reproducing its genes are threatened by death, Hart and Burns (2012) extended Nairne's et al. (2007) work to consider the possible conceptual relation between processing of death and processing of survival (survival can be conceptually viewed as the avoidance of death); they suggested a functional analogy between the memorial benefits of survival-based processing and the memorial benefits of processing of one's death. That is, given that dying is a chief threat to any organism's chances of reproduction, thoughts of death may enhance the encoding and retention of information that could be used to directly or indirectly extend lifespan and enhance reproductive fitness. In their first study, they demonstrated that when compared with several control conditions (e.g., watching TV group or toothache group), priming for death produced a better score at the recall stage (Hart and Burns, 2012; Burns et al., 2014b). In their following studies, the survival-processing scenarios and death-processing scenarios were directly compared, and results indicated that both scenarios produced similar recall performance. Therefore, they suggested that death-related thoughts may function in a manner similar to survival-related thoughts in enhancing recall, which was termed as the “dying to remember” (DTR) effect (Burns et al., 2014b).

Other studies have also examined this issue more critically, reaching different conclusions concerning the influence of death in the survival effect (Klein, 2014). Specifically, Klein argued that there are profound conceptual and functional differences between thoughts of death and thoughts of survival. His results showed that although death-related thoughts could promote high levels of recall, the level achieved did not match that produced by survival-related thoughts: essentially, participants in the survival group performed better than those in the death group. He also suggested that survival- and death-related thoughts might rely on different mechanisms to achieve their effects. A more recent paper summarized the controversy on this topic, concluding that the idea that survival's mnemonic effect was due to activation of thoughts of death was not readily supported, though there was some overlap between survival and death processing (Bugaiska et al., 2015).

Although memory researchers have conducted several studies to investigate the mechanisms underlying the adaptive memory effect, finding that death and survival processing are partially the same (Hart and Burns, 2012; Burns et al., 2014b; Klein, 2014), unfortunately, the “survival-” orienting task and the “death-” orienting task were adopted in different paradigms in previous studies. Specifically, the phenomenon of “survival enhancing retention,” revealed by Nairne et al. (2007), was observed in a processing task, in which participants were oriented to process words in terms of its relevance of survival value. However, the DTR effect reported by Hart and Burns (2012) was observed in a priming task, in which participants first thought about death and then processed words regarding their pleasantness without encoding their survival or death value. It is conceivable that the effect of priming on recall, as observed in DTR studies, differs from the effect of processing during survival-related tasks (Bugaiska et al., 2015). Although some studies have directly compared the survival-processing scenarios with the death-processing scenarios and found both to produce similar recall levels (Burns et al., 2014a), to our knowledge, no study to date has directly compared the survival-priming scenarios with the death-priming scenarios.

Furthermore, the pleasantness rating task was often selected as a control task and served as a baseline in previous adaptive memory research. This is not problematic if the experimental condition is a survival/death priming task. However, there might be a mismatch between the control task and the experimental task if the experimental condition is a survival/death-processing task. This is because the pleasantness rating task does not typically induce relational processing but only item-specific processing. Specifically, item-specific processing refers to the encoding of items' individual characteristics, while relational processing refers to the encoding of relationships between items. As rating words for their relevance to a specific scenario may induce both item-specific and relational processing (Burns et al., 2013), relevance rating may cause a higher proportion of recall than pleasantness rating merely as a result of the difference in encoding depth.

To conclude, although memory researchers have conducted several studies to investigate the mechanisms underlying the adaptive memory processing effect, finding partial similarity of death and survival processing (Hart and Burns, 2012; Klein, 2014), the question of whether death scenarios improve recall as much as survival scenarios, regardless of the processing/priming paradigms, remains unsolved. Therefore, in Experiment 1, we tested whether death scenario improve recall by as much as survival scenario, both in a processing task and priming task. Experiment 2 was designed to replicate Experiment 1 and directly compared the survival-priming scenarios with the death-priming scenarios.

## Experiment 1

### Method

#### Ethics statement

This study was approved by the Southwest University ethics review board. Prior to obtaining written informed consent from each participant, a complete explanation of the study was provided.

#### Participants

A total of 159 undergraduates at Southwest University (Chongqing, China), who were paid 10 yuan for their time, participated in this experiment. Participants were randomly assigned to one of six groups. Participants' demographic information is presented in Table 1. They were tested in individual sessions lasting approximately 25 min.

**Table 1:** Demographic information in each group in experiment 1.

	**Processing**			**Priming**		
	**Survival**	**Death**	**Control**	**Survival**	**Death**	**Control**
Number of participants	23	24	24	30	30	28
Number of Females	16	16	15	18	17	16
Mean age (SD)	21.52(1.59)	21.46(1.69)	21.38(1.53)	21.20(1.35)	21.47(1.41)	22.14(1.43)

#### Stimuli

To build the word list, we collected 200 words, the meanings of which were not highly relevant to either survival or death (all nouns: e.g., snow, pencil, moon). These words were then presented to an independent sample of 25 participants, who were asked to rate their relevance to survival and death, the valence, and the familiarity of words on a 1–7 scale. Finally, we selected a subset of 40 words that were well-matched on their relevance to survival and death (mean = 2.45 for survival and mean = 2.29 for death). In general, these words were rated as neither positive nor negative (mean = 4.37), and as familiar to participants (mean = 4.74). All participants viewed the same list of 40 stimulus words in a random order. The list of items is shown in the Appendix.

#### Procedure

A 2 (task type: processing or priming) × 3 (scenario: survival, death, or control) between-subjects design was used. Participants in the processing groups were asked to rate the relevance of each word to a corresponding scenario depending on the group to which they were assigned, while participants in the priming groups were required to rate the pleasantness of each word instead.

Participants were first informed that they would be seated in front of a computer and be given a word-rating task. Then, the specific instructions to each group were announced. To match the death scenario with the survival scenario used by Nairne et al. (2007) and Klein (2012), we used the instructions and procedure as follows:

**Survival processing:** “In this task, I would like you to imagine that you are stranded on the desolate grasslands, suffering from hunger and cold. You did your best to find steady supply of food and water and protect yourself from predators.” Finally rescue teams saved your life, and you survived. “I am going to show you a list of words, and I would like you to rate how relevant each of these words would be for you in this survival situation. Some words may be relevant and others may not, it is up to you to decide. You must use a rating scale of 1 (totally irrelevant) to 5 (extremely relevant).”
**Death processing:** “In this task, I would like you to imagine that you are stranded on the desolate grasslands, suffering from hunger and cold. You couldn't find steady supply of food and water and protect yourself from predators.” Rescue teams were too late to help you, and you died. “I am going to show you a list of words, and I would like you to rate how relevant each of these words would be for you in this death situation. Some words may be relevant and others may not, it is up to you to decide. You must use a rating scale of 1 (totally irrelevant) to 5 (extremely relevant).”
**Control processing**: In this task, I would like you to imagine that you are stranded on the grasslands. I am going to show you a list of words, and I would like you to rate how relevant each of these words would be for you in this grasslands situation. “Some words may be relevant and others may not, it is up to you to decide. You must use a rating scale of 1 (totally irrelevant) to 5 (extremely relevant).”
**Survival priming:** “In this task, I would like you to imagine that you are stranded on the desolate grasslands, suffering from hunger and cold. You did your best to find steady supply of food and water and protect yourself from predators.” Finally rescue teams saved your life, and you survived. “Please briefly describe the emotions that the thought of your survival arouses in you; Jot down, as specifically as you can, what you think will happen to you as you physically survived.” I am going to show you a list of words, and I would like you to rate the pleasantness of each word. “Some of them may be pleasant and others may not, it's up to you to decide. You must use a rating scale of 1 (extremely unpleasant) to 5 (extremely pleasant).”
**Death priming:** “In this task, I would like you to imagine that you are stranded on the desolate grasslands, suffering from hunger and cold. You couldn't find steady supply of food and water and protect yourself from predators.” Rescue teams were too late to help you, you died. “Please briefly describe the emotions that the thought of your death arouses in you; Jot down, as specifically as you can, what you think will happen to you as you physically died.” I am going to show you a list of words, and I would like you to rate the pleasantness of each word. “Some of them may be pleasant and others not, it's up to you to decide. You must use a rating scale of 1 (extremely unpleasant) to 5 (extremely pleasant).”
**Control priming:** “In this task, I would like you to imagine that you are stranded on the grasslands. Please briefly describe the emotions arouses in you; Jot down, as specifically as you can, what you think will happen to you as you physically in this situation.” I am going to show you a list of words, and I would like you to rate the pleasantness of each word. “Some of them may be pleasant and others not, it's up to you to decide. You must use a rating scale of 1 (extremely unpleasant) to 5 (extremely pleasant).”

Participants had 5–10 min to jot down their thoughts in the priming scenarios. They were asked to stop writing when 10 min had past. If they finished in less than 5 min, we encouraged them to think and write more until 5 min had elapsed. After the priming session, participants were instructed to undertake a calculation task, which lasted approximately 3 min, as a distraction task. On its completion, participants progressed to the rating task, in which each word stayed on the screen for 5 s. No mention was made of a later retention test. After the last word was rated on the computer, participants were instructed to undertake another calculation task, which lasted for nearly 3 min, as a distraction task. Subsequently, participants were required to write down the earlier rated words, in any order, using a pen and paper response sheet. They were allowed 5 min for this task. Finally, participants completed the Positive Affect and Negative Affect Schedule (PANAS; Watson et al., 1988).

### Results and discussion

The PANAS scores were presented in Table 2. A 2 (task type: processing or priming) × 3 (scenario: survival, death, or control) ANOVA showed no significant effect for the positive affect score, *p*s > 0.066, while there was a significant main effect of scenario for the negative affect (NA) score, *F*_(2, 153)_ = 4.78, *p* = 0.01, η_*p*_^2^ = 0.059, suggesting that both the survival scenario (*p* = 0.05) and the death scenario (*p* = 0.003) induced more negative emotions than the control scenario did. Neither the main effect of task type nor its interaction with scenario on NA score was significant, *ps* > 0.533.

**Table 2:** Descriptive data of Positive and Negative Affect Schedule (PANAS) in experiment 1.

	**Processing**			**Priming**		
	**Survival**	**Death**	**Control**	**Survival**	**Death**	**Control**
Positive affect	2.98(0.71)	2.80(0.64)	2.89(0.80)	2.75(0.81)	2.50(0.77)	2.78(0.64)
Negative affect	1.83(0.62)	2.01(0.85)	1.49(0.60)	1.88(0.78)	1.99(0.71)	1.67(0.56)

**Figure 1:**
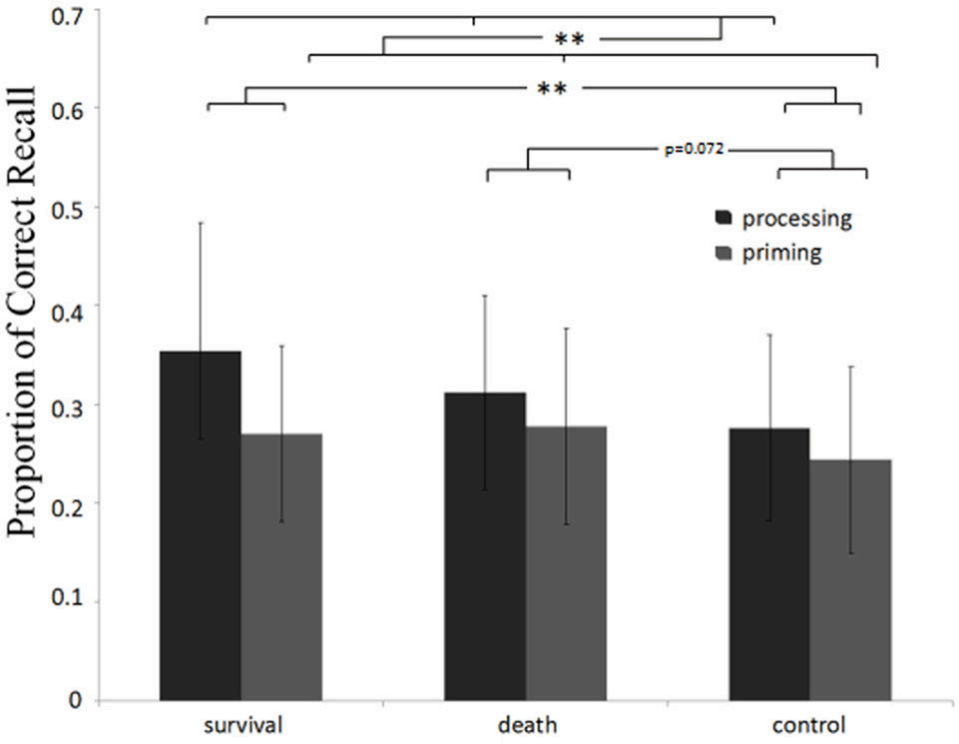
Average proportion of correct recall sorted by conditions in Experiment 1. Error bars indicate standard deviations in each group. ^**^p < 0.01.

The average proportions of correct recall in the six groups are presented in Figure 1. The result showed that task type had a significant effect, *F*_(1, 153)_ = 9.82, *p* = 0.002, η_*p*_^2^ = 0.060, with the recall rate higher in the processing condition than that in the priming condition. On the differences between scenarios, we also found a significant effect, *F*_(2, 153)_ = 3.66, *p* = 0.028, η_*p*_^2^ = 0.046. Specifically, participants in the survival scenario recalled more words than those in the control scenario, *p* = 0.009; while those in the death scenario had a better recall than those in the control scenario at a marginally significant level, *p* = 0.072. However, there was no difference between the death and survival scenarios, *p* > 0.394. The interaction effect between scenario and task type was not significant, *F*_(2, 153)_ = 1.12, *p* = 0.327, η_*p*_^2^ = 0.015. Moreover, including NA as a covariate did no change the main effect of scenarios, *F*_(2, 152)_ = 3.55, *p* = 0.031, η_*p*_^2^ = 0.045.

To examine whether the recall advantage could be attributed to disproportionate recall of words rated as more or less pleasant/relevant, we computed the proportions of recall for the words rated at each point on the 1–5 pleasantness/relevance rating scale (see Table 3). A 3 (scenario: survival, death, or control) × 5 (rating: 1, 2, 3, 4, or 5) × 2 task type (task type: processing or priming) ANOVA showed no scenario × rating interaction either in the processing paradigm, *F*_(8, 272)_ = 1.15, *p* = 0.328, η_*p*_^2^ = 0.033, or in the priming paradigm, *F*_(8, 340)_ = 1.57, *p* = 0.132, η_*p*_^2^ = 0.036.

**Table 3:** Mean proportions of words recalled as a function of experimental condition and pleasantness/relevance rating in Experiment 1.

		**Pleasantness//Relevance Ratings**				
		**1 (extremely unpleasant)**	**2**	**3**	**4**	**5 (extremely pleasant)**
Priming	Survival	0.52(0.37)	0.40(0.30)	0.26(0.20)	0.25(0.15)	0.28(0.26)
	Death	0.34(0.31)	0.26(0.21)	0.22(0.16)	0.29(0.17)	0.34(0.26)
	Control	0.38(0.27)	0.27(0.22)	0.21(0.14)	0.24(0.12	0.31(0.30)
Processing	Survival	0.32(0.10)	0.27(0.16)	0.30(0.20)	0.33(0.20)	0.39(0.25)
	Death	0.28(0.16)	0.35(0.28)	0.29(0.31)	0.39(0.19)	0.46(0.30)
	Control	0.29(0.32)	0.16(0.13)	0.28(0.25)	0.38(0.21)	0.35(0.32)

In sum, we found that both the survival scenario and the death scenario enhanced recall regardless of the processing/priming paradigms, and that the effect could not be explained by disproportionate retention of items rated as pleasant/relevant (or unpleasant/irrelevant), or by affect. Moreover, participants' proportion of correct recall did not show a difference between the survival scenario and the death scenario, suggesting that the mechanisms for favoring survival and death in memory performance seem to rely on similar, rather than different processes.

Nevertheless, the instruction of the control scenario was less detailed than that of the other two scenarios in Experiment 1, leaving an open possibility that it was the detail of scenario that improved memory. Moreover, due to the apparently small recall differences among the three priming groups in Experiment 1, Experiment 2 was designed to replicate Experiment 1 and test whether the death scenario improved recall as much as the survival scenario in the priming task using a more detailed control scenario. Additionally, in Experiment 2 we also added questions designed to assess the extent of thinking of survival and death during priming to ensure that the survival and death scenarios specifically induced survival- and death-related thoughts, respectively.

## Experiment 2

### Method

#### Ethics statement

This study was approved by the Southwest University ethics review board. Prior to obtaining written informed consent from each participant, a complete explanation of the study was provided.

#### Participants

Eight-three healthy Chinese college students participated in this study as paid volunteers. Two participants failed to complete all measures, leaving 81 participants for data analysis. 26 participants were randomly assigned to the survival scenario (14 females, mean age = 20.34 years, *SD* = 1.54 years); 28 to the death scenario (15 females, mean age = 20.58 years, *SD* = 1.85 years); and 27 to the control scenario (14 females, mean age = 20.25 years, *SD* = 1.50 years). Participants gave their written informed consent prior to participation, and they were tested in individual sessions lasting approximately 25 min.

#### Stimuli

The 40 stimulus words were identical to those of Experiment 1.

#### Procedure

A one-factor experimental design with scenario (survival, death, or control) as a between-subject variable was used. The procedure of Experiment 2 was similar to that of Experiment 1 with the exception that (1) the processing paradigms were not adopted, (2) the instructions for the control scenario were more detailed than that in Experiment 1, (3) questions designed to assess the extent of thinking of survival and death during priming were included. The instructions for the detailed control scenario are presented below.

**Control priming:** “In this task, I would like you to imagine that you are stranded on the grasslands. The land is flat and open, and the temperature is suitable. There are boundless green pastures in the distance. There are white and flock of cattle and sheep near here. Please briefly describe the emotions arouses in you; Jot down, as specifically as you can, what you think will happen to you as you physically in this situation.” I am going to show you a list of words, and I would like you to rate the pleasantness of each word. “Some of them may be pleasant and others not, it's up to you to decide. You must use a rating scale of 1 (extremely unpleasant) to 5 (extremely pleasant).”

After the priming session, participants were instructed to report the extent of thinking of survival and death on an 11-point scale (e.g., “To what extent did you think of ‘death’/ ‘survival’ after answering two questions? 0 = not at all; 10 = very much).”

### Results and discussion

Participants' subjective reports (thinking of death and thinking of survival) are shown in Table 4. A one-way ANOVA with scenario (survival, death, or control) as a between-subject variable revealed that participants in the death scenario reported more thinking of death compared with both those in the survival scenario (*p* < 0.001) and in the control scenario (*p* < 0.001), *F*_(2, 78)_ = 78.41, *p* < 0.001, η_*p*_^2^ = 0.668. In addition, participants in the survival scenario reported more thinking of survival compared with those in the death scenario (*p* = 0.003) and those in the control scenario (*p* = 0.006), *F*_(2, 78)_ = 5.71, *p* = 0.005, η_*p*_^2^ = 0.128.

**Table 4:** Descriptive data of thoughts of death and thoughts of survival in experiment 2.

	**Priming**		
	**Survival**	**Death**	**Control**
Thinking of death	5.10(2.78)	7.59(2.12)	0.44(1.28)
Thinking of survival	7.64(1.71)	5.34(2.88)	5.48(3.41)

**Figure 2:**
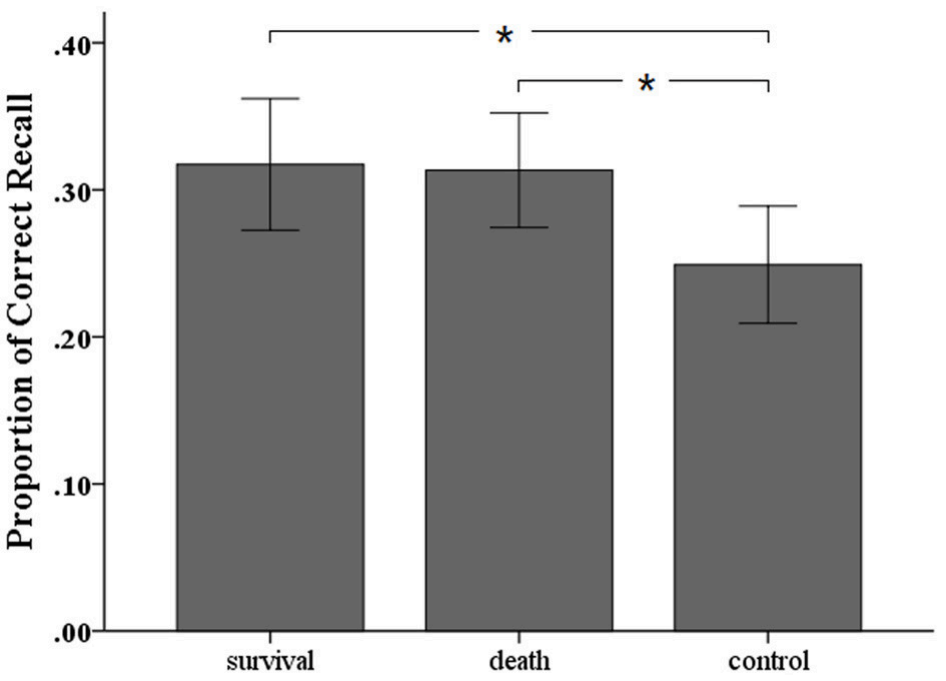
Average proportion of correct recall sorted by different priming conditions in Experiment 2. Error bars indicate standard deviations in each group. ^*^p < 0.05.

The average proportions of correct recall in the three groups were presented in Figure 2. One-way ANOVA showed that there was a significant effect of scenario, *F*_(2, 78)_ = 3.65, *p* = 0.030, η_*p*_^2^ = 0.086, suggesting that participants recalled more words both in the survival scenario (*p* = 0.019) and in the death scenario (*p* = 0.025) than in the control scenario. However, there was no difference between the death scenario and the survival scenario, *p* = 0.890, 1-β = 0.53.

To examine whether the recall advantage could be attributed to disproportionate recall of words rated as more or less pleasant, we computed the proportions of recall for the words rated at each point on the 1–5 pleasantness rating scale (see Table 5). A 3 × 5 ANOVA showed no scenario (survival vs. death vs. control) × pleasantness interaction, *F*_(8, 312)_ = 1.55, *p* = 0.138, η_*p*_^2^ = 0.038.

**Table 5:** Mean proportions of words recalled as a function of experimental condition and pleasantness rating in Experiment 2.

	**Pleasantness Ratings**				
	**1 (extremely unpleasant)**	**2**	**3**	**4**	**5 (extremely pleasant)**
Survival	0.54(0.30)	0.38(0.29)	0.26(0.16)	0.24(0.15)	0.32(0.33)
Death	0.47(0.29)	0.38(0.34)	0.36(0.22)	0.34(0.30)	0.34(0.30)
Control	0.45(0.19)	0.27(0.25)	0.24(0.15)	0.42(0.36)	0.42(0.36)

As anticipated, the survival scenario induced more thoughts of survival than the death and control scenarios did. A similar analysis revealed that death-related thoughts were reported significantly more often in the death scenario than in either the survival or control scenario. In sum, these results suggested that our scenario setting (comparing death priming to survival priming) successfully separated thoughts of death from thoughts of survival.

Moreover, we replicated the results of Experiment 1 and found that participants' proportion of correct recall showed no difference between the survival and the death scenario, while participants in both conditions recalled more words than those in the control scenario, even when the instruction for the control scenario was as detailed as that for the survival/death scenarios. Accordingly, our data suggested that both survival and death scenarios improve recall relative to the control scenario in a priming task, and that the effect could not be easily explained by the elaboration or scenario details or by disproportionate retention of items rated as pleasant (or unpleasant).

## Discussion

In sum, we found that participants' correct recall proportion showed no difference between the survival and the death condition, while participants in both conditions recalled more words than those in the control condition, implying that the mechanisms for favoring survival and death in memory performance seem to rely on similar rather than different processes. Moreover, the correct recall proportion was significantly higher in the processing condition than that in the priming condition, indicating that relevance rating leads to a higher proportion of recall than does pleasantness rating.

Since Nairne and colleagues adopted a functionalist view of memory systems (Nairne et al., 2007), the field of adaptive memory research has flourished and accumulated a large body of literature over the last decade, including studies focusing on the adaptive memory mechanisms underlying the enhancing effects of death-related thoughts and survival-related thoughts on recall (Hart and Burns, 2012; Burns et al., 2014a; Klein, 2014; Bugaiska et al., 2015). These studies draw different conclusions about whether the mechanisms underlying the DTR effect and survival effect overlap. Consequently, we highlight Burns et al.'s (2014a) suggestion that more research on this issue is needed. The current study provides further evidence that, compared to a well-matched control condition, both survival-related thoughts and death-related thoughts improved memory recall, as shown by the similar effect of the survival scenarios and death scenarios, in both processing and priming tasks.

The tasks used in previous studies to directly compare survival-processing scenarios with death-processing scenarios could be all categorized as consciousness-driven. For example, Bell et al. (2013) compared a situation in which participants were required to imagine that they had decided to commit suicide with a situation that involved survival at grasslands; participants rated words for their usefulness in such a situation. Moreover, Klein (2014) compared the traditional survival condition, in which participants rated the relevance of words to this survival situation, with a death condition, in which participants rated the relevance of words to their demise. Similarly in Burns et al.'s (2014a) study, participants' tasks were consciously related to the scenarios. Although studies by Hart and Burns (2012) and Burns et al. (2014b) have explored a death priming protocol, in which participants first thought about death and then rated the pleasantness of unrelated words, to our knowledge, no prior study has directly compared survival priming scenarios with death priming scenarios on an unconscious level. The results of our experiment showed that both the survival priming scenario and the death priming scenario enhanced people's memory retention compared to the control priming condition, which is in line with Burns and Hart, and Bugaiska's arguments that the mechanisms responsible for survival processing and death processing overlap. Moreover, we are the first to extend the aforementioned studies' findings to a more general application, showing that the enhancement of memory by the mind's evolution is not only present at a conscious level but also at an unconscious level.

Our results also show that the processing task improved correct recall proportion more than did the priming task, suggesting that the relational rating task caused a higher recall proportion than did the pleasantness rating task, which is known to merely induce item-specific processing. Though it has been suggested that survival tasks may encourage processing of both item-specific and relational information whereas control tasks may only involve the former (Nairne et al., 2008; Burns et al., 2011), our findings suggest that it was inappropriate to use the pleasantness rating as a control condition if the experimental condition emphasizes relational processing (rating the relevance of words to the survival/death scenarios). This is because the mismatch in processing style (relational vs. item-specific) between the experimental condition and the control condition would enlarge the difference in memory performance between them. Thus, we could speculate that the difference between the survival scenario and the control condition would be larger when the control task is item-specific (e.g., pleasantness rating) rather than relational (e.g., picnic-planning), just as Klein (2012) presented in his study. This idea is also consistent with the findings in Burns et al.'s (2011) study, suggesting that when the control task engaged both item-specific and relational processing, the survival processing advantage was eliminated.

Some limitations of this study and directions for future work in this area must be noted. First, our results for the processing task were somewhat inconsistent with some previous studies in which participants who processed stimuli in the death scenario recalled less than those in the survival scenario (Klein, 2014). We cautiously speculate that the mismatch between the survival scenario and the death scenario in their study caused this difference. For example, Klein (2014) simply instructed participants to imagine dying in the death scenario, while participants were instructed to imagine being “stranded on the desolate grasslands, suffering from hunger and cold, trying to find a steady supply of food and water and protect themselves from predators” in the survival scenario. To address this previous study's deficiency, the current study modified the instructions of the death scenario and the control scenario to more closely align them with the classic survival scenario. Second, although we report similar effects of the survival and death scenarios, we did find some differences between them, showing that compared to the control condition, the survival scenarios significantly improved people's memory retention, while the death scenarios only marginally improved it. As we have cautiously matched both scenarios between conditions to make them similarly thematic, detailed and concrete, and matched the items to make them relevant to neither survival nor death, we would not attribute these differences to mismatch between stimuli. Third, we speculated that the “processing task” and “priming task” differed in consciousness, with the processing paradigm task operating at the conscious level, while the priming paradigm task operates at the unconscious level. However, it should be noted that consciousness was not directly manipulated or measured in the current study. Future study should seek to remedy this limitation.

In short, our results supported our hypothesis that mortality-salience and survival scenarios result in comparable recall performance, in both processing and priming tasks. Our findings extend the adaptive memory literature and highlight a similar mechanism underlying death-related and survival-related memory enhancement effect.

## Author contributions

XinZ, HL, and JY designed the experiment. XiaZ and HL conducted the experiment and analyzed the data by supervision of JY. HL, XiaZ, and JY wrote the manuscript.

### Conflict of interest statement

The authors declare that the research was conducted in the absence of any commercial or financial relationships that could be construed as a potential conflict of interest.

## Appendix

### Experimental stimuli

**Table:**

画家	(painter)	探戈	(tango)	手稿	(manuscript)	把手	(handle)	灰尘	(dust)
柜子	(cabinet)	海报	(poster)	花纹	(streak)	蓝图	(blueprint)	邮件	(mail)
旗帜	(flag)	昆虫	(insect)	幻想	(fantasy)	王后	(queen)	磁带	(tape)
香水	(perfume)	英国	(England)	木材	(timber)	建筑	(building)	标题	(title)
橡皮	(eraser)	气味	(smell)	柱子	(pillar)	熨斗	(iron)	乐队	(band)
铅笔	(pencil)	泡沫	(froth)	僧侣	(monk)	雪花	(snow)	国王	(king)
月亮	(moon)	报纸	(newspaper)	头发	(hair)	寺庙	(temple)	斑点	(spot)
足球	(football)	绯红	(scarlet)	演员	(performer)	奖牌	(medal)	诗人	(poet)
